# Gut Microbiota-Targeted Intervention of Hyperlipidemia Using *Monascus*-Fermented Ginseng

**DOI:** 10.3390/ph18050661

**Published:** 2025-04-30

**Authors:** Qing Zhou, Cuiting Yang, Mingyue Jia, Qingsong Qu, Xinhui Peng, Weishuo Ren, Guoqing Li, Yueyang Xie, Bingxuan Li, Xinyuan Shi

**Affiliations:** 1 School of Chinese Materia Medica, Beijing University of Chinese Medicine, Yangguang South Street, Beijing 102488, China; zhouqing2286@163.com (Q.Z.); quqingsong@outlook.com (Q.Q.); pengxinhui1999@163.com (X.P.); 13821830107@163.com (W.R.); lishiyi0803@163.com (G.L.); xyyjiayou0824@163.com (Y.X.); 2002@163.com (B.L.); 2 School of Life Science, Beijing University of Chinese Medicine, Yangguang South Street, Beijing 102488, China; cuitingy2022@163.com (C.Y.); 15092148063@163.com (M.J.); 3 Key Laboratory for Production Process Control and Quality Evaluation of Traditional Chinese Medicine, Beijing Municipal Science &Technology Commission, Beijing 100029, China

**Keywords:** gut microbiota, fecal microbiota transplantation, fermentation, hyperlipidemia, ginseng

## Abstract

**Background/Objectives:** Hyperlipidemia (HLP) encompasses a spectrum of poorly understood lipid metabolism disorders that are frequently overlooked or misdiagnosed, potentially leading to multiple complications. While the gut microbiota has been implicated in HLP pathogenesis, the causal relationships and molecular mechanisms remain elusive. This study aimed to investigate the therapeutic mechanisms of *Monascus*-fermented ginseng (MFG) on HLP through gut microbiota modulation and explore treatment potential via fecal microbiota transplantation (FMT). **Methods:** The MFG-modulated gut microbiota was transplanted into HLP mice. Systemic evaluations, including serum biochemical parameter detection, histopathological section analysis, 16S rRNA sequencing, and fecal metabolomics, were conducted to assess therapeutic efficacy and identify associated metabolic pathways. **Results:** FMT significantly improved lipid profiles, reduced body weight, and attenuated hepatic lipid accumulation in HLP mice. Mechanistically, it enhanced cholesterol excretion and fatty acid β-oxidation while suppressing lipogenic regulators, concurrently promoting primary-to-secondary bile acid conversion. Gut microbiota analysis revealed that the MFG intervention effectively normalized the Firmicutes/Bacteroidetes ratio and enriched beneficial microbiota. **Conclusions:** These findings demonstrate FMT’s therapeutic value in HLP management and provide new perspectives on utilizing fermented herbal medicines for metabolic disorders via gut microbiota reprogramming.

## 1. Introduction

Hyperlipidemia (HLP), a metabolic disorder characterized by dysregulated lipid homeostasis and excessive adiposity, constitutes a major pathogenic determinant of metabolic syndrome. Substantial clinical evidence has established its strong association with life-threatening cardiovascular sequelae, including atherosclerosis [1], cerebrovascular accidents [2], coronary artery disease [3], and acute myocardial infarction [4]. While current therapeutic regimens involving hypolipidemic agents such as acipimox and simvastatin demonstrate clinical efficacy, their chronic administration is frequently accompanied by iatrogenic complications [5]. This therapeutic dilemma underscores the urgent need for developing intervention strategies that achieve optimal lipid modulation while minimizing procedural invasiveness.

Traditional Chinese Medicine (TCM), characterized by its time-honored pharmacological legacy in dyslipidemia management, represents a vast repository of phytochemical candidates with demonstrated hypolipidemic potential [6]. Phytochemical analyses and clinical observations reveal that *Monascus*-fermented rice (Hongqu) mediates marked reductions in total cholesterol (TC), triglycerides (TG), and low-density lipoprotein cholesterol (LDL-C), establishing its therapeutic candidacy for statin-intolerant patients exhibiting statin-associated myopathy or hepatotoxicity [7]. Furthermore, a series of studies have demonstrated that poria [8], ginseng [9], magnolia [10], Pericarpium *Citri reticulatae* [11], gynostemma [12], Rhizoma *Polygonati odorati* [13], cassia seed [14], etc., have excellent therapeutic effects on HLP. Microbial fermentation has been recognized in TCM since ancient times for its potential to enhance the quality of the medicinal substances present in the aforementioned medicinal plants. Numerous studies have demonstrated that fermentation could augment the anti-HLP properties of ginseng [15]. For instance, the fermented *Eucommia ulmoides leaves* have been shown to mitigate HLP by maintaining intestinal homeostasis and modulating gut microbiota [16]. Our previous research has indicated that *Monascus*-fermented ginseng (MFG) could improve various indices in HLP mice and exert regulatory effects on gut microbiota [17]. These findings suggest that fermented TCM formulations may offer a promising strategy for improving lipid metabolism.

According to extant research, the treatment of HLP with fermented TCM is associated with the modulation of the gut microbiota [18]. The gut microbiota constitutes a complex ecosystem that plays a critical role in host metabolism, immunity, and overall health [19]. The disordered gut microbiota is closely related to the development of disease [20]. The main external factors that can affect the composition of the gut microbiota in generally healthy people include antibiotic therapy and major dietary changes. Recent studies demonstrated that dietary factors affect gut homeostasis at multiple levels, influencing both intestinal cells and the gut microbiota [21]. Therefore, the involvement of the gut microbiota in disease processes and the potential to modify the gut microbiota through external factors make microbiota-targeted interventions a promising avenue for the development of new therapeutic approaches.

Increasing evidence suggests that the pathogenesis of HLP is intricately linked to the structural and functional alterations in the gut microbiota, with dysbiosis being recognized as a hallmark of metabolic disorders [22]. HLP can cause changes in the gut environment, which can lead to dysbiosis of the gut microbiota in the body. Studies have shown that children and adolescents with HLP show lower levels of acetic, propionic, and butyric acids in their feces, which are associated with a decrease in short-chain fatty acid-producing bacteria such as *Akkermansia*, *Bacteroides*, *Roseburia*, and *Faecalibacterium* [23]. Consequently, investigating the anti-HLP effects and the underlying mechanisms of fermented TCM in regulating the gut microbiota has emerged as a significant research focus [24]. The mechanisms underlying the regulation of HLP by the gut microbiota are intricate and multifaceted. Both the composition of the gut microbiota and its metabolites significantly influence host physiology [25]. Numerous animal studies have demonstrated that gut microbiota-derived metabolites interact with specific receptors to modulate the host’s lipid metabolism [26]. For instance, bile acids (BAs) [27] and short-chain fatty acids (SCFAs) [28] have been shown to regulate hepatic lipid and glucose levels, thereby maintaining energy homeostasis. Therefore, the modulation of the gut microbiota and its metabolites may represent a novel therapeutic target for the alleviation of HLP.

Fecal microbiota transplantation (FMT) is potentially an effective treatment for HLP that restores the gut microbiota to normalcy. FMT involves the extraction of beneficial gut microbiota from healthy individuals and its subsequent transplantation into patients with dysbiosis to stabilize their gut microbiota and treat their various diseases [29]. FMT has been employed in the clinical management of *Clostridium difficile* infection, inflammatory bowel disease, metabolic syndrome, diabetes, autism, multiple sclerosis, Parkinson’s disease, and cancer [30]. Studies have demonstrated that the gut microbiota of high-fat diet (HFD) mice is influenced by resveratrol supplementation and can regulate lipid metabolism, promote the development of beige adipocytes within white adipose tissue, reduce inflammation, and enhance the intestinal barrier function [31]. Recent clinical trials demonstrate that HLP patients receiving FMT from lean donors exhibit improved LDL-C profiles and hepatic steatosis indices, though the mechanistic underpinnings remain poorly understood [32].

In our initial study, we transplanted the gut microbiota altered through the MFG intervention into germ-free mice and observed several effects, including the regulation of SCFAs and BAs metabolism, lipid reduction, and the amelioration of liver injury. While germ-free mice offer valuable insights for research in preventive, diagnostic, therapeutic, and translational medicine concerning disease-associated microbiomes and targets, notably, they exhibit significant physiological and morphological differences compared to conventional mice [33]. Therefore, FMT should be investigated in common mice in subsequent clinical applications.

In this study, the MFG-intervened gut microbiota was transplanted into SPF-grade HLP mice to examine its role and underlying mechanisms in the context of FMT treatment for HLP. The investigation employed a range of indicators, including markers related to lipid metabolism, liver tissue morphology, the expression of genes associated with lipid metabolism, and the metabolism of SCFAs and BAs in feces, as well as the composition of gut microbiota. The findings are intended to provide a theoretical foundation for the development of personalized microbial therapies for the management of HLP.

## 2. Results

### 2.1. Effects of FMT on Phenotype and Biochemical Indicator Alterations in Mice

**Figure 1:**
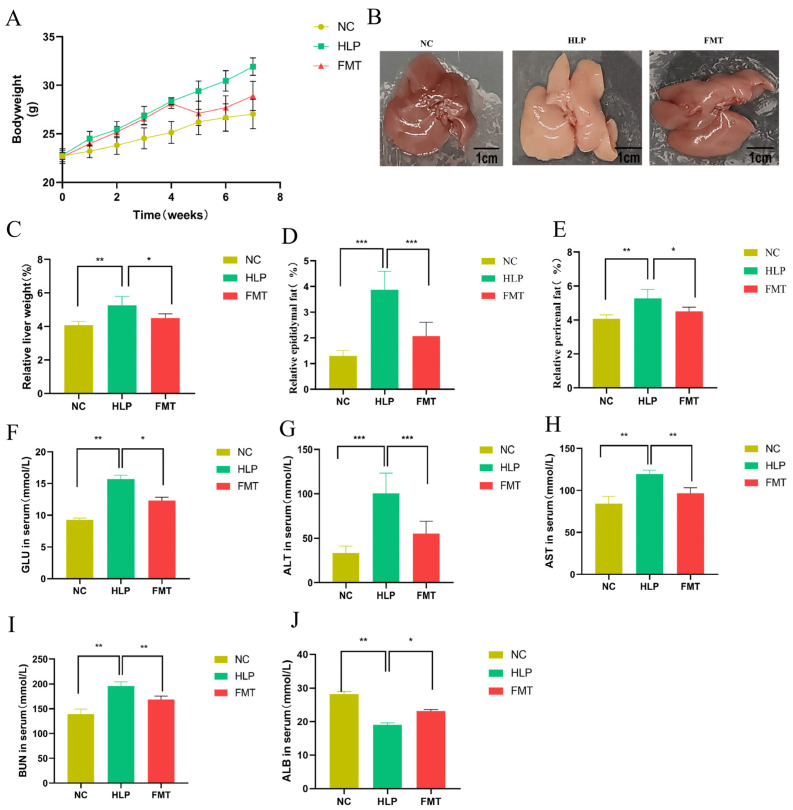
Effect of FMT on phenotype and biochemical indicator alterations: (**A**) body weight, (**B**) liver performance, (**C**) liver weight, (**D**) epididymal fat, (**E**) perirenal fat, (**F**) blood glucose, (**G**) serum ALT, (**H**) serum AST, (**I**) serum BUN, and (**J**) serum ALB. * *p* < 0.05; ** *p* < 0.01; *** *p* < 0.001.

After FMT, mice in the HLP group exhibited the highest body weight (Figure 1A), liver weight (Figure 1C), epididymal fat weight (Figure 1D), and perirenal fat weight (Figure 1E), with these metrics being significantly different from those observed in the NC and FMT groups (*p* < 0.05). The livers in the HLP group appeared whitish and greasy, with a notable increase in liver volume. Conversely, in the FMT group, the livers gradually regained their red coloration and glossy appearance (Figure 1B). FMT demonstrated a capacity to modulate these indicators to varying extents, indicating that the MFG-intervened gut microbiota may inhibit lipid overaccumulation and partially restore its phenotype.

ALT and AST are recognized as sensitive biomarkers for hepatocyte damage, with their levels in serum exhibiting a positive correlation with the severity of hepatic injury. BUN serves as an indicator of glomerular filtration function, while ALB, the most abundant plasma protein, shows decreased serum levels in response to renal injury. In comparison to the HLP group, the FMT group demonstrated significantly lower levels of GLU, ALT, AST, and BUN, alongside the significantly elevated serum levels of ALB (Figure 1F–J) (*p* < 0.05). These findings revealed that FMT was effective in mitigating HLP induced by an HFD and in partially restoring hepatic and renal function.

### 2.2. Effects of FMT on Lipid Accumulation in Mice

**Figure 2:**
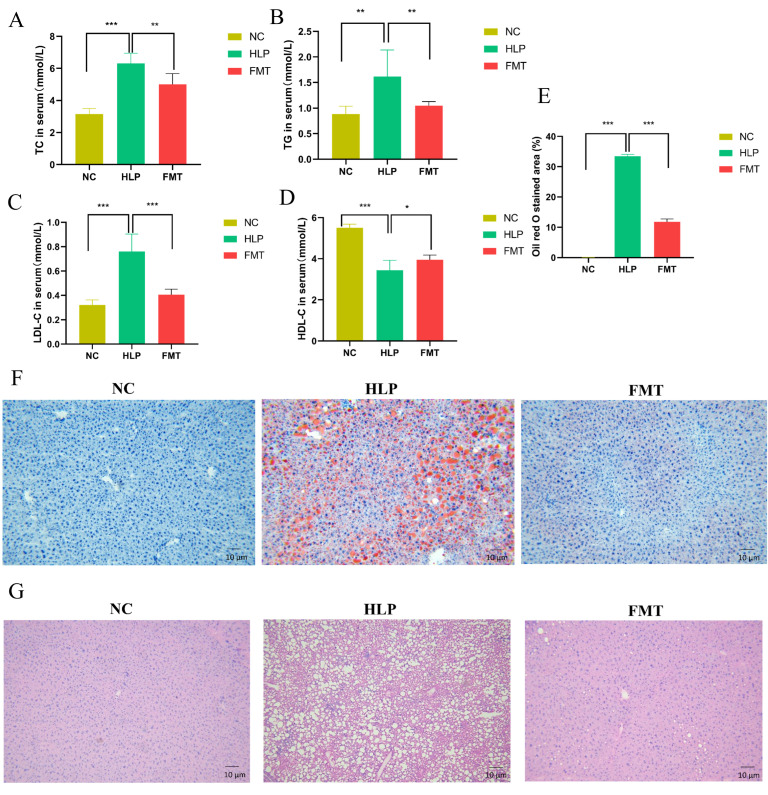
Effects of FMT on lipid metabolism. (**A**–**D**) Serum lipid profile: (**A**) total cholesterol, (**B**) triglycerides, (**C**) low-density lipoprotein cholesterol, and (**D**) high-density lipoprotein cholesterol. (**E**–**G**) Hepatic histopathological analysis: (**E**) quantification of oil red O-stained lipid droplets, (**F**) representative microphotographs of oil red O, and (**G**) hematoxylin and eosin (**E**) staining of liver tissue. * *p* < 0.05; ** *p* < 0.01; *** *p* < 0.001.

Blood samples from mice were collected to analyze serum lipid profiles, including TC, TG, LDL-C, and HDL-C. Among the three groups, the HLP group exhibited the highest levels of TC, TG, and LDL-C, as well as the lowest level of HDL-C (Figure 2A–D). In comparison to the HLP group, the FMT group demonstrated a significant reduction in TC, TG, and LDL-C levels and an increase in HDL-C levels (*p* < 0.05). These findings suggest that FMT could markedly improve serum cholesterol distribution and ameliorate HLP in mice.

The lipid droplet area in the liver tissue sections of mice in the HLP group was 33.47 ± 0.63%, whereas in the FMT group, it was 11.86 ± 0.93% (Figure 2E). Furthermore, hepatocytes in the HLP group exhibited structural alterations, including disorganization, severe steatosis, and inflammatory cell infiltration. Following FMT, there was a significant reduction in the number of lipid droplet vacuoles and inflammatory infiltration in liver tissues, and the structure of liver lobules was restored (Figure 2F,G). The results suggest that the MFG-intervened gut microbiota could significantly reduce fat accumulation and restore the histomorphology of the liver in HLP mice.

### 2.3. Effects of FMT on Hepatic Gene Expression Profiles in Mice

**Figure 3:**
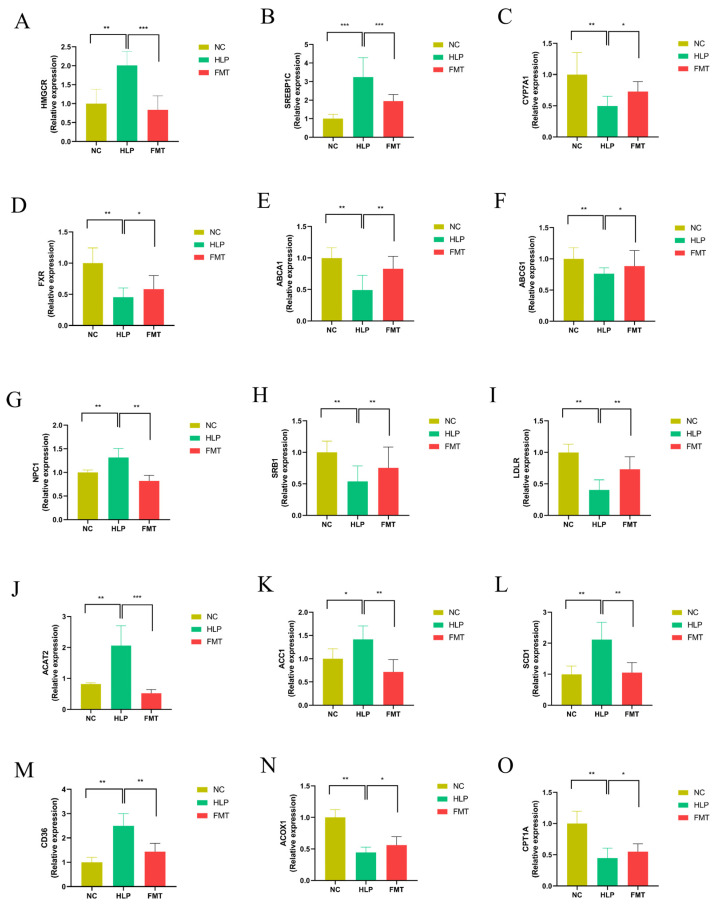
Effects of FMT on hepatic gene expression profiles: (**A**) *Hmgcr*, (**B**) *Srebp1c*, (**C**) *Cyp7a1*, (**D**) *Fxr*, (**E**) *Abca1*, (**F**) *Abcg1*, (**G**) *Npc1*, (**H**) *Srb1*, (**I**) *Ldlr*, (**J**) *Acat2*, (**K**) *Acc1*, (**L**) *Scd1*, (**M**) *Cd36*, (**N**) *Acox1*, and (**O**) *Cpt1a*. * *p* < 0.05; ** *p* < 0.01; *** *p* < 0.001.

The expression of genes associated with TC production was significantly downregulated in the FMT group compared to the HLP group, including the hydroxymethylglutarate monoacyl coenzyme A reductase (*Hmgcr*) gene and the sterol regulatory element-binding protein 1C (*Srebp1c*) gene. Concurrently, the 7α-hydroxylase (*Cyp7a1*) gene, which catalyzes the synthesis of BAs from cholesterol for absorption by the small intestinal mucosa, was significantly upregulated. In contrast, the farnesol-derived X receptor (*Fxr*) gene, which negatively regulates this process, was significantly downregulated (Figure 3A–D). In contrast, the expression of the ATP-binding cassette transporter protein A1 (*Abca1*) and ATP-binding cassette transporter protein G1 (*Abcg1*) genes, which are implicated in the accelerated transport of excess TC, was significantly upregulated. Conversely, the expression of the Niemann–Pick type C1 (*Npc1*) gene, known for its role in inhibiting this transport, was significantly downregulated (Figure 3E–G). Additionally, the genes scavenger receptor type B1 (*Srb1*) and low-density lipoprotein receptor (*Ldlr*), which are involved in TC esterification and the prevention of TC accumulation, exhibited significant upregulation, whereas the expression of acyl coenzyme A–cholesterol acyltransferase 2 (*Acat2*), an enzyme that inhibits this process, was significantly downregulated (Figure 3H–J). These findings suggest that the gut microbiota modified by the MFG intervention could mitigate TC synthesis and TC absorption in HLP mice. This effect was achieved by downregulating the expression of genes involved in TC synthesis and absorption, while upregulating genes associated with TC excretion, thereby facilitating the translocation and excretion of TC from the liver, ultimately exerting lipid-lowering effects.

In comparison to the HLP group, the FMT group exhibited a significant downregulation in the expression of genes related to fatty acid production and uptake, including the acetyl-CoA carboxylase 1 (*Acc1*), stearoyl-CoA desaturase 1 (*Scd1*), and cluster of differentiation 36 (*Cd36*) genes (Figure 3K–M). Genes implicated in fatty acid oxidative catabolism, such as Acyl-CoA oxidase 1 (*Acox1*) and carnitine palmitoyl transferase 1 (*Cpt1a*) (Figure 3N,O), exhibited significant upregulation. These findings suggest that the gut microbiota modulated by the MFG intervention may contribute to the reduction in free cholesterol synthesis and absorption and enhance their oxidative catabolism in HLP mice. This is achieved through the downregulation of genes associated with free cholesterol synthesis and absorption, and the upregulation of genes related to free cholesterol oxidative catabolism, ultimately leading to lipid-lowering effects.

### 2.4. Effects of FMT on Altered Gut Microbiota in Mice

**Figure 4:**
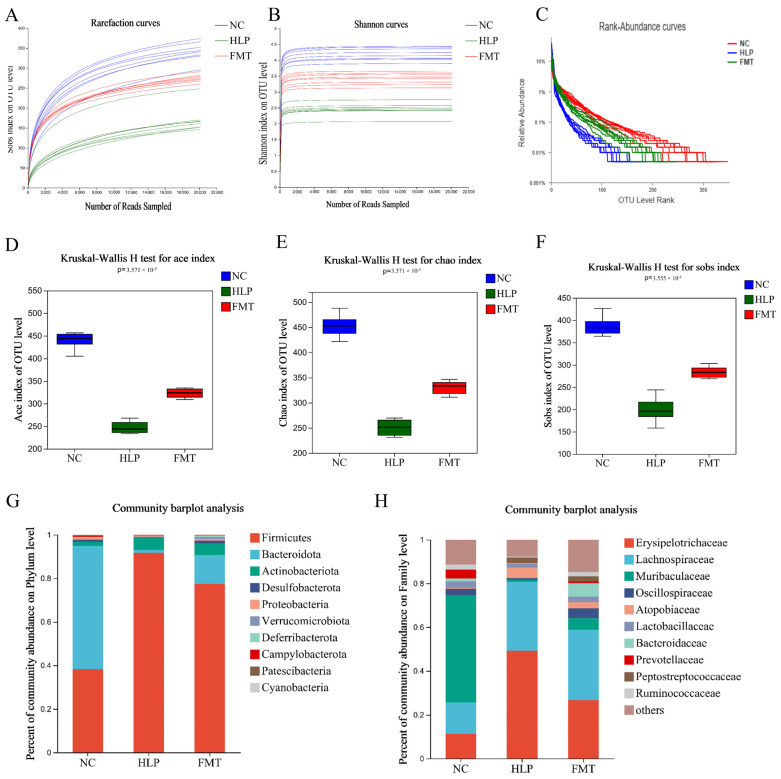
Effects of FMT on altered gut microbiota: (**A**) rarefaction curves, (**B**) Shannon curves, (**C**) Rank–Abundance curves, (**D**) ACE index, (**E**) Chao index, (**F**) Sobs index, (**G**) percent of community abundance on phylum level, and (**H**) percent of community abundance on family level.

Using the Illumina MiSeq sequencing platform (Miseq PE300/NovaSeq PE250, accessed on 23 March 2025) to target the V3–V4 region of the 16S rRNA gene, a total of 1,663,800 effective sequences were obtained from 30 samples, averaging 69,325 sequences per group. Rarefaction curves indicated that the sequencing depth was sufficient to cover all species present in the samples. Additionally, the Rank–Abundance curve demonstrated that, compared to the HLP group, the FMT group exhibited improved diversity and evenness (Figure 4A–C). Furthermore, the MFG-intervened gut microbiota restored the reduced ACE, Chao, and Sobs indices observed in HLP mice (Figure 4D–F). The data suggest that the community abundance and homogeneity of gut microbiota were enhanced in HLP mice following FMT, thereby confirming that the MFG-intervened gut microbiota can augment the α diversity of gut microbiota in HLP mice.

To investigate the alterations in gut microbial composition resulting from the MFG-intervened gut microbiota interventions, bacterial abundances at various taxonomic levels were assessed. As illustrated in Figure 4G, eleven dominant phyla (including Firmicutes, Bacteroidota, and Actinobacteriota) were shared among all groups, albeit with differing relative abundances. Numerous studies have suggested that an elevated Firmicutes/Bacteroidetes (F/B) ratio is correlated with obesity [34]. In the NC group, the relative abundance of Firmicutes and Bacteroidota was 38.00% and 56.70%, respectively. In the HLP group, these values were 91.60% and 1.43%, respectively. In the FMT group, the relative abundance of Firmicutes and Bacteroidota was 77.40% and 13.30%, respectively. These results indicate that the MFG-intervened gut microbiota could significantly reduce the value of F/B in the gut microbiota of HLP mice, suggesting that it can play a therapeutic role in HLP by regulating gut microbiota [35]. At the family level, the HLP group demonstrated a higher relative abundance of Erysipelotrichaceae and a lower relative abundance of Muribaculaceae, Lactobacillaceae, Oscillospiraceae, Bacteroidaccae, Prevotellaceae, and Ruminococcaceae, in comparison to the NC group (Figure 4H). However, the MFG-intervened gut microbiota significantly restored the relative abundances of these bacteria compared with the gut microbiota of the HLP group. In a study examining non-alcoholic fatty liver disease in mice, it was observed that an increased abundance of Erysipelotrichaceae was associated with the accumulation of pro-inflammatory compounds, thereby exacerbating the inflammatory response in the liver [36]. The Oscillospiraceae have been linked to conditions such as obesity, gallstones, and chronic constipation [37]. The Lactobacillaceae family, commonly found in fermented foods and Lactobacillus-fortified dairy products, is well known for its probiotic properties [38]. Research indicates that bacteria from the Lactobacillaceae family may alleviate diabetes symptoms and regulate obesity by modulating adipokine expression. In comparison to the HLP group, the FMT group exhibited a significant increase in Oscillospiraceae, Bacillaceae, and Lactobacillaceae, suggesting that the gut microbiota intervention with MFG could ameliorate HLP by upregulating these bacterial families. HLP altered the gut microbial composition of mice, and FMT partially restored the healthy composition of gut microbes disrupted by HFD. These findings imply that the MFG-mediated modulation of gut microbiota composition may offer a therapeutic approach to treating HLP.

### 2.5. Effects of FMT on SCFAs Contents in Mice

**Figure 5:**
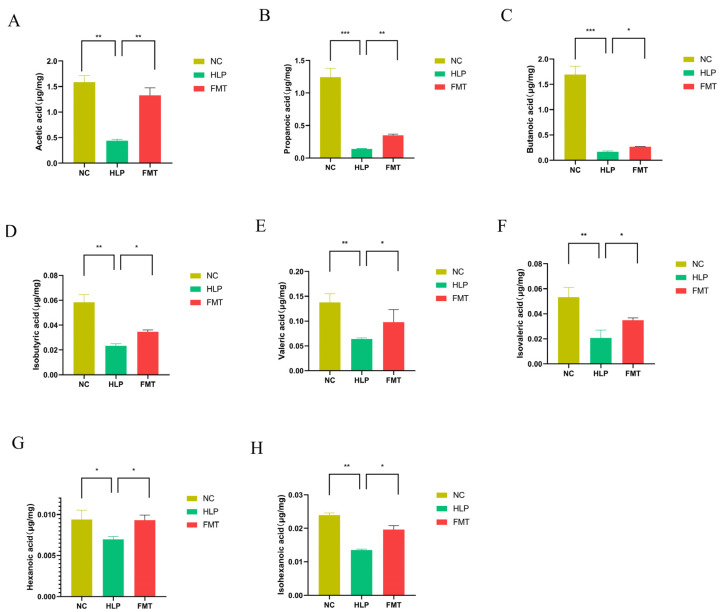
Effects of FMT on SCFA contents: (**A**) acetic acid, (**B**) propionic acid, (**C**) butyric acid, (**D**) isobutyric acid, (**E**) valeric acid, (**F**) isovaleric acid, (**G**) hexanoic acid, and (**H**) isohexanoic acid. * *p* < 0.05; ** *p* < 0.01; *** *p* < 0.001.

As the end metabolites of the microbial fermentation of carbohydrates in the gut, SCFAs play an important role in lipid metabolism and participate in multiple lipid metabolism pathways. It was found that FMT significantly elevated the levels of acetic acid, propionic acid, butyric acid, and isobutyric acid compared to those of the HLP group (Figure 5A–D). In addition, the analysis of valeric, isovaleric, hexanoic, and isohexanoic acids showed significantly lower levels of valeric acid in the feces of mice of the HLP group, which were restored in mice of the FMT group (Figure 5E–H). These findings suggest that the MFG-intervened gut microbiota regulated the SCFAs metabolism in mice.

### 2.6. Effects of FMT on the Regulation of BAs Metabolism in Mice

**Figure 6:**
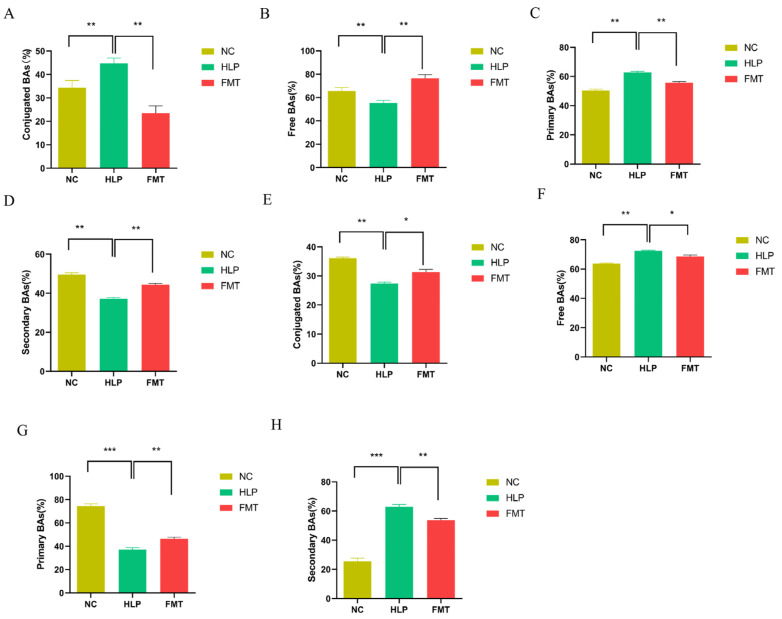
Effects of FMT on regulation of BAs metabolism: (**A**) conjugated BAs in serum, (**B**) free BAs in serum, (**C**) primary BAs in serum, (**D**) secondary BAs in serum, (**E**) conjugated BAs in feces, (**F**) free BAs in feces, (**G**) primary BAs in feces, (**H**) secondary BAs in feces. * *p* < 0.05; ** *p* < 0.01; *** *p* < 0.001.

**Figure 7:**
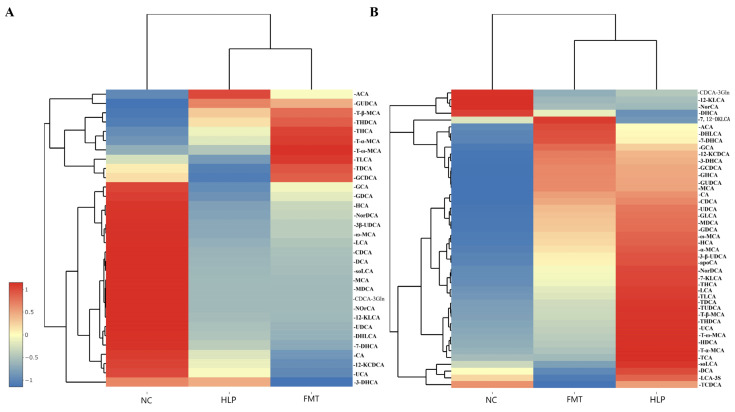
Heat map of BAs: (**A**) in serum and (**B**) in feces.

Compared to the NC group, the HLP group exhibited elevated serum concentrations of BAs, whereas the FMT group demonstrated significantly reduced levels of conjugated BAs. This pattern was also evident in the concentrations of primary BAs. Conversely, the FMT group showed significantly higher concentrations of free BAs and secondary BAs relative to the NC group (Figure 6A–D). The HLP group exhibited lower concentrations of bound BAs in fecal matter, whereas the FMT group demonstrated significantly higher concentrations of conjugated BAs compared to the NC group. A similar trend was observed in the concentrations of primary BAs. However, the FMT group showed elevated levels of free BAs and secondary BAs relative to the NC group (Figure 6E–H). Compared to the NC group, the HLP group exhibited significantly reduced serum levels of glycocholic acid, glycochenodeoxycholic acid, ursodeoxycholic acid, deoxycholic acid, glycodeoxycholic acid, etc., as well as decreased fecal levels of α-muricholic acid, lithocholic acid, chenodeoxycholic acid, cholic acid, 12-ketodeoxycholic acid, etc. However, following the transplantation of MFG-intervened gut microbiota, there was a notable increase in BA levels (Figure 7). These findings suggest that MFG may regulate BA species in both serum and feces, thereby exerting a therapeutic effect on HLP in mice.

## 3. Discussion

HLP is implicated in the pathogenesis of numerous metabolic disorders, necessitating the urgent identification of effective therapeutic interventions. Accumulating evidence reveals that the gut microbiota and its metabolites play important roles in HLP [39]. Based on the current understanding of gut microbiota, people rebuild the host’s disturbed gut microbiota through microbiota transplantation to restore the normal and stable state of the host’s gut microbiota and maintain the normal intestinal state of the host [40]. FMT is potentially an effective treatment for HLP that restores the gut microbiota to normalcy. Previous studies have shown that MFG has a therapeutic role in HLP and modulates the gut microbiota composition of HLP mice; however, the efficacy of FMT via the MFG-intervened gut microbiota is unknown. Consequently, this study focused on the therapeutic efficacy and mechanism of FMT for HLP by applying the MFG-intervened gut microbiota.

HFD feeding effectively induced HLP in mice, with symptoms aligning with those documented in the relevant literature [41]. A prolonged elevation of TG or TC significantly increases the risk of stroke, atherosclerosis, and cardiovascular disease [42]. Conversely, maintaining normal levels of HDL-C facilitates reverse cholesterol transport, promoting cholesterol degradation, thereby reducing the likelihood of cardiovascular disease [43]. The MFG-intervened gut microbiota led to improved serum lipid profiles, reduced body weight, and decreased lipid deposition in HLP mice. Additionally, a recovery of liver damage and alterations in the expression of genes related to hepatic lipid metabolism were observed.

The lipid metabolic disorder associated with HLP is characterized by an increase in lipogenesis and a decrease in lipolysis. *Acc1* and *Scd1* are critical downstream proteins in the fatty acid de novo synthesis pathway [44]. These enzymes facilitate the synthesis of fatty acids and contribute to TG accumulation in the liver. Specifically, *Acc1* plays a pivotal role in fatty acid biosynthesis and represents a potential target for anti-obesity and lipid-lowering therapies [45]. In contrast, *Scd1* functions as a rate-limiting enzyme in fatty acid production. In HLP patients, *Scd1* levels rise, and inhibiting *Acc1* and *Scd1* gene expression reduces liver fatty acid production. *Acox1* and *Cpt1a* enhance fatty acid β-oxidation, boosting lipid metabolism [46]. *Cd36*, which regulates fatty acid uptake via dynamic palmitoylation, is significantly more expressed in HLP patients compared to healthy individuals [47]. This study revealed that the MFG-intervened gut microbiota significantly downregulates *Acc1*, *Scd1*, and *Cd36* genes while upregulating *Acox1* and *Cpt1a* genes compared to those in the HLP group. This shift reduces fatty acid synthesis and absorption and enhances fatty acid oxidation, potentially decreasing HLP-induced liver fat accumulation.

The composition of gut microbiota plays a significant role in the pathogenesis of HLP and serves as a crucial target for the development of lipid-lowering therapeutics. Interventions targeting the gut microbiota, such as those involving MFG, have the potential to significantly alter the microbial composition associated with HLP. Fecal microbiota analysis has identified specific bacterial taxa, such as the decreased abundance of Erysipelotrichaceae, which are positively associated with hyperlipidemia and obesity [48]. Research has demonstrated the effects of formulations containing Lactobacillaceae on adiposity, microbiota alteration, and the metabolome in mice fed an HFD [49]. Notably, many genera within the Oscillospiraceae family can produce SCFAs, such as butyrate, and have been identified as potential candidates for next-generation probiotics [50]. Members of the Bacteroidaceae family contribute to the breakdown of food and the production of nutrients and energy for the body. They are also involved in the fermentation of carbohydrates, utilization of nitrogenous substances, and biotransformation of bile acids and other steroids. The metabolites secreted by various anabolic bacteria help maintain immune system stability and are the primary producers of SCFAs in the human gut [51]. Considering the changes in the fecal microbiota after MFG treatment, we hypothesized that the increased abundance of Muribaculaceae, Lactobacillaceae, Oscillospiraceae, Bacteroidaccae, Prevotellaceae, and Ruminococcaceae might be a key step for MFG to exert its function through the fecal microbiota.

Notably, the modulation of lipid metabolism by gut microbiota through BAs and SCFAs is a well-documented mechanism in lipid-lowering research. The transplantation of MFG-intervened gut microbiota resulted in a significant increase in fecal SCFAs, as well as a significant increase in serum and fecal BAs, and HLP-induced metabolic disturbances associated with BAs were restored. This is in line with other studies looking at the metabolism of SCFAs and BAs during HLP treatment [52]. The dynamic interconversion between BAs and TC plays a pivotal role in the regulation of lipid metabolism and the maintenance of TC homeostasis within the body [53]. *Fxr* is an essential nuclear receptor for BAs and modulates the synthesis and homeostasis of BAs by regulating the expression of *Cyp7a1* in the liver [54]. *Abca1*, *Abcg1*, and *Srb1* are three other important transporters for hepatic transport and excretion of TC, which accelerate TC efflux and prevent intracellular accumulation [55]. This study shows that the MFG intervention activated the expression of *Cyp7a1*, *Fxr*, *Abca1*, *Abcg1*, and *Srb1* in the liver compared to those in the HLP group. Crucially, FMT significantly inhibited Hmgcr and *Srebp1c* genes, the key genes for TC synthesis in HLP mice, reducing liver TC accumulation and enhancing its movement to extrahepatic areas.

It has been discovered that SCFAs play an important role in human health through the gut microbiota–host lipid metabolism axis [56]. The concentration of SCFAs could be significantly increased through FMT. Research has demonstrated that SCFAs can prevent obesity, hyperinsulinemia, hypertriglyceridemia, and hepatic steatosis in mouse models [57]. The ameliorative effects of gut microbiota on HLP and fatty liver in mice subjected to the MFG intervention may be partially attributed to the elevated levels of SCFAs. Consequently, the MFG-intervened gut microbiota could serve as a potent modulator, ameliorating hepatic cholesterol accumulation by regulating BAs and SCFAs metabolism.

Furthermore, there was an increase in the richness and α diversity of gut microbiota, changes in the structure of gut microbiota, an increase in beneficial bacteria, and a decrease in harmful bacteria after FMT. The gut microbiota plays a crucial role in drug metabolism, energy metabolism, and immune response. In this study, the observed reduction in intestinal bacteria in the HLP group of mice aligns with findings from previous research, resulting in metabolic disorders such as dyslipidemia and impaired glucose tolerance [58]. Bacteroides, which are prevalent probiotics, contribute to the breakdown of food and the production of essential nutrients and energy for the body. They are involved in the fermentation of carbohydrates and the biotransformation of BAs and other steroids [59]. Our study found a significant increase in bacteroidetes in the FMT group compared to the HLP group. Daniostataceae bacteria, which produce endotoxins and contribute to intestinal inflammatory diseases, were significantly reduced after fecal microbiota transplantation. This indicates that the MFG intervention can regulate gut microbiota in HLP mice, lowering lipids by increasing beneficial bacteria and reducing harmful ones.

FMT is the latest mode of gut microbiota manipulation for the treatment of metabolic disease, which seems to be more effective than other existing approaches to microbiota manipulation [60]. As evidenced by several animal-based studies, FMT could effectively improve the manifestations of non-alcoholic fatty liver disease [61]. There has been an increase in the number of clinical trials evaluating the role of FMT in obesity treatment [62]. Therefore, in this study, we collected, processed, and transplanted fecal samples from mice receiving the MFG intervention into SPF-grade HLP mice in order to investigate and validate the efficacy and potential mechanisms of FMT for the treatment of HLP at the in vivo level. This study offers novel insights into the identification of therapeutic targets of TCM for HLP, as well as the clinical prevention and treatment of HLP using TCM. However, due to temporal and logistical constraints, the specific bacterial genera involved have not yet been identified. Future research could address this limitation through colony isolation and pure bacterial transplantation.

## 4. Materials and Methods

### 4.1. Materials

Ginseng aged more than five years was purchased from Shanghai Ruiling Biotechnology Co. (Shanghai, China). *Monascus* (strain number: CICC 40942) was purchased from the China Center of Industrial Culture Collection (Wuhan, China). Total RNA Extraction Kit was purchased from Tiangen Biochemical Technology Co., Ltd. (Beijing, China). RNase-free water was purchased from Beijing Lambride Trading Co., Ltd. (Beijing, China). The corresponding primers were synthesized by Sangon Biotech (Shanghai, China). Biochemical kits were sourced from Nanjing JianCheng Bioengineering Institute (Nanjing, China).

MFG preparation: The preparation of MFG was performed by formerly reported methods [17]. A *Monascus* liquid seed solution was inoculated into ginseng root homogenate with a 3% inoculum, fermented at 30 °C and 160 rpm for 6 days, freeze-dried, and powdered.

### 4.2. Animal Experiments

Male SPF-grade C57BL/6J mice (5 weeks old, 20–25 g) were obtained from Beijing Speford Biotechnology Co., Ltd. (Beijing, China). The mice were housed under standard conditions, which included a temperature of 25 ± 2 °C, a humidity of 60 ± 5%, and a 12-h light/dark cycle, with free access to food and water. All animal study protocols were approved by the Laboratory Animal Management Committee of Beijing University of Chinese Medicine (approval code: BUCM-4-2021091306-2125; approval date: 22 May 2021).

**Figure 8:**
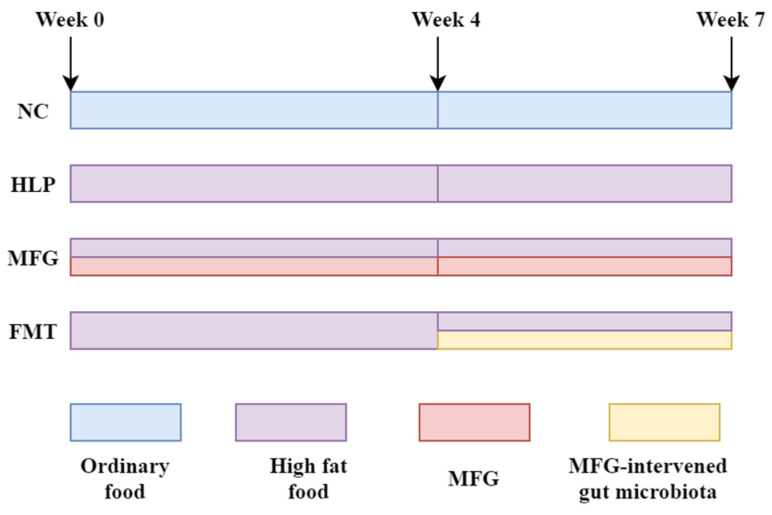
Animal experiment flow.

The flow of animal experiments is presented in Figure 8. Mice were randomly assigned to four groups (*n* = 10 each):

(1) NC group: normal chow diet.
(2) HLP group: HFD (4.2% fat, 20% protein, 52% carbohydrate, 2.3% fiber).
(3) MFG group: HFD + MFG (2 g·kg^−1^·d^−1^, oral gavage).
(4) FMT group: HFD + treated with the feces from the mice in the MFG group (daily from week 4).

Following 28-day interventions, animals underwent humane euthanasia via CO_2_ asphyxiation. Serum and hepatic tissues were immediately collected, weighed (precision ± 0.1 mg), snap-frozen in liquid N_2_, and archived at −80 °C for subsequent analyses.

### 4.3. Fecal Microbiota Transplantation

Fresh fecal specimens from MFG-treated mice were aseptically collected in sterile polypropylene tubes and immediately processed under anaerobic conditions (85% N_2_, 10% H_2_, 5% CO_2_). Homogenization was performed in pre-reduced PBS (0.01 M, pH 7.4) at a 1:10 (*w*/*v*) ratio using a vortex mixer. The homogenate underwent serial filtration through stainless mesh filters (75 μm, 38 μm, 25 μm; Millipore, Burlington, MA, USA) followed by centrifugation (300× *g*, 4 °C, 1 min). The microbe-enriched supernatant was aliquoted into cryovials under an anaerobic atmosphere. For FMT, each recipient mice received 200 μL of freshly prepared bacterial suspension via oral gavage daily for 28 consecutive days. Each dose contained 10^8^ CFU·mL^−1^ viable bacteria as quantified by the bacterial count.

### 4.4. Serum Biochemical Indicators and Staining Analysis

The serum levels of TC, TG, LDL-C, high-density lipoprotein cholesterol (HDL-C), alanine aminotransferase (ALT), aspartate aminotransferase (AST), blood urea nitrogen (BUN), albumin (ALB), and glucose (GLU) were measured using enzymatic assay kits following the manufacturer’s instructions. For liver histological analysis, fresh liver samples were fixed in 10% paraformaldehyde, embedded in paraffin, and then sliced into 5 μm thick sections. These sections were stained with hematoxylin–eosin (H&E) and oil red O, and digital images were captured using a Nikon Eclipse E100 microscope (Nikon, Tokyo, Japan).

### 4.5. Quantitative Real-Time PCR (RT-qPCR)

Total RNA was extracted from the liver using an RNA extraction kit. RNA integrity was verified with 1% agarose gel electrophoresis (RIN > 8.0) and quantified using NanoDrop 2000 (Thermo Scientific, Waltham, MA, USA). First-strand cDNA synthesis was performed with 1 μg total RNA using PrimeScript™ RT Master Mix in 20 μL reactions containing 5 × RT buffer, dNTP mix (10 mM each), RNase inhibitor (40 U/μL), and oligo (dT) 20/random hexamer primers (50 μM). RT-qPCR was conducted on a QuantStudio 6 Flex system with SYBR Green I chemistry. Reactions (20 μL) contained the following: 10 μL of 2 × SYBR Premix Ex Taq™ II, 0.8 μL of each primer (10 μM), 2 μL of cDNA template, and 6.4 μL of nuclease-free water. Thermocycling parameters were as follows: initial denaturation: 95 °C for 30 s; 40 cycles: 95 °C for 5 s and 60 °C for 34 s; melt curve: 95 °C → 65 °C (0.5 °C/5 s increment). Gene expression was normalized to β-actin using the comparative 2^−ΔΔCt^ method. Primer sequences used in this study are all shown in Supplementary Table S1.

### 4.6. BAs Analysis

The BAs profiling of fecal and serum samples was conducted via UPLC-MS/MS (Waters BEH C_18_ column; mobile phase: 0.1% formic acid/methanol) with ESI ± ionization (3.0 kV, 100–1000 *m*/*z*, 70 FWHM). Information on the ion-pair parameters of various BAs is shown in Supplementary Table S2. Fecal aliquots (50 mg) were subjected to methanol/water (4:1) extraction with cryogenic homogenization (4 °C → −10 °C, 50 Hz), ultrasonication (5 °C, 40 kHz), and centrifugation (13,000 rpm, 4 °C). For serum BAs, 100 μL of the sample solution was aspirated and centrifuged, and the supernatant was evaporated under nitrogen. Subsequently, 100 μL of 50% acetonitrile/water solution was added for re-dissolution, mixed thoroughly, and then centrifuged at 4 °C for 15 min at 12,000 rpm. The resulting supernatant was used for machine detection. MS detection utilized dd-MS^2^ (17,500 FWHM) with automated quantitation via the software MassHunter (v10.0.707.0, Agilent, Santa Clara, CA, USA accessed on 23 March 2025) against the calibration curves (Supplementary Table S3).

### 4.7. SCFAs Analysis

SCFAs were analyzed using GC-MS (Agilent 8890B/5977B, Beijing, China, EI source 70 eV) with an HP-FFAP column (He carrier, 1 mL/min) with the following temperature gradient: 80 °C (1 min) → 120 °C @40 °C/min → 200 °C @10 °C/min → 230 °C (3 min). Samples (25 mg) were cryogenically ground in 0.5% H_3_PO_4_, followed by extraction with n-butanol (IS, 10 μg/mL) and centrifugation (13,000 rpm, 4 °C). From the supernatant, a 400 µL volume was transferred to a centrifuge tube, mixed with 0.2 mL of *n*-butanol containing the internal standard, vortexed for 10 s, subjected to low-temperature ultrasound for 10 min, and centrifuged again. The supernatant was then transferred to sample vials for analysis. Instrument parameters included a split injection (1 μL, 10:1), an inlet temperature of 180 °C, an ion source at 230 °C, and a quadrupole at 150 °C. Compounds were identified and quantified using the software MassHunter, with the mass spectrum peak area used to calculate concentrations based on a standard curve (Supplementary Table S4).

### 4.8. 16S rRNA Sequencing

Fresh fecal samples were collected, and the total genomic DNA was extracted using an E.Z.N.A. Soil DNA Kit. After confirming the quality of DNA samples through agarose gel electrophoresis, the extracted DNA samples were used as the templates to amplify the V3−V4 hypervariable regions of the 16S rRNA gene using the primers 338F (5′-ACTCCTACGGGAGGCAGCAG-3′) and 806R (5′-GGACTACHVGGGTWTCTAAT-3′). After the amplicons were purified and quantified, the pair-end library was constructed following Illumina’s genomic DNA library preparation procedures, and high-throughput sequencing was performed on a MiSeq Illumina Sequencing Platform (Miseq PE300/NovaSeq PE250). After filtering and merging the obtained reads via FLASH (http://www.cbcb.umd.edu/software/flash, version 1.2.7, accessed on 23 March 2025), the operational taxonomic units (OTUs) were clustered and annotated at 97% similarity using the software Uparse (http://drive5.com/uparse/, version 7.1, accessed on 23 March 2025). All representative reads were annotated and blast-searched against the Silva database using the RDP classifier (http://rdp.cme.msu.edu/, version 2.2, accessed on 23 March 2025) with a confidence threshold of 70%.

### 4.9. Statistical Analysis

Graphical illustrations were performed with GraphPad Prism 8.0. Statistical analysis was performed with one-way ANOVA tests using the software SPSS (version 19.0, SPSS, Chicago, IL, USA). *p* < 0.05 was considered statistically significant.

## 5. Conclusions

In conclusion, this study elucidated the disruption patterns of gut–liver structures and functions in HLP and demonstrated how FMT can restore the homeostasis of gut microbiota and metabolites to ameliorate HLP. However, the therapeutic effects observed in SPF-grade HLP mice represent an essential but preliminary step in understanding microbiota–host interactions in lipid metabolism disorders. Therefore, it is critical to conduct rigorous validation in human-relevant systems—such as humanized mice, organoids, or patient-derived microbiota—to systematically assess the efficacy of FMT in HLP prior to clinical translation. In addition, mechanistic studies and translational exploration might prove to be an important area for future research, for example, the mechanistic analysis of host–microbiota interactions using gut–liver organoid co-cultures. These investigations will systematically reveal the therapeutic potential of microbiota modulation while maintaining scientific rigor in translational research.

## Data Availability

The sequence data of this study have been deposited in the NCBI database (https://www.ncbi.nlm.nih.gov), accessed on 4 September 2024 and their accession number is PRJNA1156384.

## Supplementary Materials

The following supporting information can be downloaded at: https://www.mdpi.com/article/10.3390/ph18050661/s1, Table S1: Primers used in this study; Table S2: Table of BA ion-pair parameters; Table S3: BAs standard curve; Table S4: SCFAs standard curve.

## Abbreviations

The following abbreviations are used in this manuscript:

HLP	hyperlipidemia
MFG	*Monascus*-fermented ginseng
FMT	fecal microbiota transplantation
TCM	Traditional Chinese Medicine
TC	total cholesterol
TG	triglycerides
LDL-C	low-density lipoprotein cholesterol
HDL-C	high-density lipoprotein cholesterol
ALT	alanine aminotransferase
AST	aspartate aminotransferase
BUN	blood urea nitrogen
ALB	albumin
GLU	glucose
BAs	bile acids
SCFAs	short-chain fatty acids
HFD	high-fat diet
RT-*q*PCR	Quantitative real-time PCR
*Hmgcr*	hydroxymethylglutarate monoacyl coenzyme A reductase
*Srebp1c*	sterol regulatory element-binding protein 1C
*Cyp7a1*	7α-hydroxylase
*Fxr*	farnesol-derived X receptor
*Abca1*	ATP-binding cassette transporter protein A1
*Abcg1*	ATP-binding cassette transporter protein G1
*Npc1*	Niemann–Pick type C1
*Srb1*	scavenger receptor type B1
*Ldlr*	low-density lipoprotein receptor
*Acat2*	acyl coenzyme A–cholesterol acyltransferase 2
*Acc1*	acetyl-CoA carboxylase 1
*Scd1*	stearoyl-CoA desaturase 1
*Cd36*	cluster of differentiation 36
*Acox1*	Acyl-CoA oxidase 1
*Cpt1a*	carnitine palmitoyl transferase 1
F/B	Firmicutes/Bacteroidetes
OTUs	operational taxonomic units
