# Supplementary material for: Gut Microbiota-Targeted Intervention of Hyperlipidemia Using Monascus-Fermented Ginseng
Source: Pharmaceuticals (Basel). 2025 Apr 30;18(5):661. doi: 10.3390/ph18050661 (PMC12114913; doi:10.3390/ph18050661)
Supplement: Supplementary file 1 [file pharmaceuticals-18-00661-s001.zip › pharmaceuticals-3573976-supplementary.pdf]

Supplementary Table S1. Primers used in this study

Primer name	Sequence (5´-3´)
ABCA1	Forward primer AACAGTTTGTGGCCCTTTTG<br>Reverse primer AGTTCCAGGCTGGCGTACTT
ABCG1	Forward primer GTGGATGAGGTTGAGACAGACC<br>Reverse primer CCTCGGGTACAGAGTAGGAAAG
ACAT2	Forward primer GAACGTGGTGGTCCATGACT<br>Reverse primer TTCAGCAGACCTCCAACCAC
ACC1	Forward primer CTCCCGATTCATAATTGGGTCTG<br>Reverse primer TCGACCTTGTTTTACTAGGTGC
ACOX1	Forward primer TTACATGCCTTTGTTGTCCCTATC<br>Reverse primer CGGTAATTGTCCATCTTCAGGTA
CD36	Forward primer CCTTACACATACAGAGTTC<br>Reverse primer CTACAGCCAGATTCAGA
CPT1A	Forward primer GATGTGGACCTGCATTCCTT<br>Reverse primer TCCTTGTAATGTGCGAGCTG
CYP7A1	Forward primer GAGGGATTGAAGCACAAGAACC<br>Reverse primer ATGCCCAGAGAATAGCGAGGT
FXR	Forward primer CTCTGTCTCCTCATCTCACCCCA<br>Reverse primer GAGAATGTGCTTCTGGGATGGT
Hmgcr	Forward primer CTCTGTCTCCTCATCTCACCCCA<br>Reverse primer GAGAATGTGCTTCTGGGATGGT
LDLR	Forward primer ACCTGCCGACCTGATGAATTC<br>Reverse primer GCAGTCATGTTCACGGTCACA
NPC1	Forward primer AACAGCGAGAGGCTCACATT<br>Reverse primer AGTGGCGTTCATGCCTGCCT
SCD1	Forward primer GGTGCCTTATCGCTTTCCTA<br>Reverse primer CCAGCCAGCCTCTTGTCTAC
SRB1	Forward primer CTTGCTGCTGAGGGAGTCTCG<br>Reverse primer CTGAAGGAGACGGAGACAGAGG
SREBP1C	Forward primer CGAACTGGGCGATGGATGAGA<br>Reverse primer TCTCCCACTTGATTGCTGACA
β-ACTIN2	Forward primer ACGTCGACATCCGCAAAGACCTC<br>Reverse primer TGATCTCCTTCTGCATCCGGTCA

Supplementary Table S2. Table of BAs ion-pair parameters

BAs name	parent ion	daughter ion	DP	CE
GCA	464.3	74	- 120	- 84
GCDCA	448.3	74.11	- 120	- 83
TCA	514.3	79.6	- 120	- 150
TCDCA	498.3	80	- 120	- 140
GUDCA	448.3	73.9	- 120	- 76
ACA	407.3	359.3	- 120	- 48
UDCA	391.3	391.1	- 120	- 50
DCA	391.3	343.2	- 120	- 50
TUDCA	498.3	79.7	- 120	- 130
HDCA	391.3	391.32	- 120	- 50
apoCA	389.2	371.2	- 120	- 40
GDCA	448.3	74.1	- 120	- 80
GLCA	432.3	74.2	- 120	- 75
α-MCA	407.3	387.31	- 120	- 48
β-MCA	407.3	389.3	- 120	- 42
7-KLCA	389.3	389.31	- 120	- 46
T-α-MCA	514.3	80.11	- 120	- 130
T-β-MCA	514.3	80.1	- 120	- 130
ω-MCA	407.3	387.3	- 120	- 48
MDCA	391.3	391.33	- 120	- 50
THDCA	498.3	79.81	- 120	- 120
THCA	514.3	79.8	- 120	- 130
TLCA	482.3	80.1	- 120	- 120
TDCA	498.3	79.8	- 120	- 120
LCA	375.3	375.31	- 120	- 50
CA	407.3	343.4	- 120	- 45
CDCA	391.3	391.3	- 120	- 50
HCA	407.3	389.4	- 120	- 45
NorCA	393.3	329.3	- 120	- 42
GHCA	464.3	74.1	- 120	- 88
NorDCA	377.3	377.3	- 120	- 49

Supplementary Table S2. Table of BAs ion-pair parameters (continued)

BAs name	parent ion	daughter ion	DP	CE
isoLCA	375.3	375.3	- 120	- 50
12-KLCA	389.3	389.3	- 120	- 43
DHLCA	373.3	373.3	- 120	- 10
LCA-3S	455.3	455.3	- 120	- 92
CDCA-3Gln	567.3	567.3	- 120	- 55
3β-UDCA	391.3	391.3	- 120	- 50
3-DHCA	405.3	405.3	- 120	- 50
CDCA-24A-βGlu	567.2	567.2	- 120	- 48
12-KCDCA	405.3	405.3	- 120	- 36
7，12-DKLCA	403.3	403.3	- 120	- 42
DHCA	401.2	401.2	- 120	- 40
UCA	407.3	407.3	- 120	- 46
7-DHCA	405.3	405.3	- 120	- 45
isoDCA	391.2	391.2	- 120	- 56
3β-CA	407.3	407.3	- 120	- 46
T-ω-MCA	514.3	514.3	- 120	- 125
CA-D4	395.3	395.3	- 120	- 50
CDCA-D4	411.3	411.3	- 120	- 45

Supplementary Table S3. BAs standard curve

BAs name	regression equation	R^2	LOQ
GCA	y = 0.16954 x - 0.00949	0.9953	0.2
GCDCA	y = 0.18148 x + 0.00138	0.9941	1.0
TCA	y = 0.15237 x + 0.00797	0.9956	0.2
TCDCA	y = 0.24110 x + 0.00157	0.9962	0.2
GUDCA	y = 0.19005 x +5.03284e^{-4}	0.9952	1.0
ACA	y = 0.05265 x + 0.00446	0.9986	0.2
UDCA	y = 0.14855 x - 0.00112	0.9972	0.5
DCA	y = 0.12313 x + 0.00634	0.9955	0.2
TUDCA	y = 0.25528 x + 0.00743	0.9933	0.5
HDCA	y = 0.14170 x + 0.01449	0.9956	0.5
apoCA	y = 0.12263 x + 2.09216e^{-4}	0.9968	0.2
GDCA	y = 0.18950 x - 0.00364	0.9964	0.2
GLCA	y = 0.24376 x + 0.01573	0.9964	2.0
α-MCA	y = 0.03938 x - 4.97576e^{-4}	0.9987	5.0
β-MCA	y = 0.01035 x - 9.08322e^{-5}	0.9931	0.2
7-KLCA	y = 0.21859 x - 0.01970	0.9973	0.2
T-α-MCA	y = 0.16800 x + 0.00142	0.9905	0.2
T-β-MCA	y = 0.22766 x + 0.02053	0.9963	1.0
ω-MCA	y = 0.02371 x + 0.00148	0.9908	0.5
MDCA	y = 0.13304 x + 0.00494	0.9931	0.2
THDCA	y = 0.40740 x + 0.00351	0.9970	0.5
THCA	y = 0.22455 x + 0.00960	0.9963	0.2
TLCA	y = 0.35788 x + 0.01244	0.9938	0.2
TDCA	y = 0.28096 x - 0.00770	0.9915	0.2
LCA	y = 0.12108 x + 0.00972	0.9946	0.5
CA	y = 0.11820 x + 0.00417	0.9934	0.5
CDCA	y = 0.09146 x + 0.00458	0.9953	0.5
HCA	y = 0.05362 x - 0.00676	0.9929	0.5
GHCA	y = 0.10717 x - 0.00156	0.9942	0.5
NorDCA	y = 0.12446 x + 0.00132	0.9963	0.5

Supplementary Table S3. BAs standard curve (continued)

BAs name	regression equation	R^2	LOQ
isoLCA	y = 0.09542 x + 0.00242	0.9935	0.2
12-KLCA	y = 0.08058 x - 0.00115	0.9939	0.2
DHLCA	y = 0.07975 x - 0.00158	0.9938	0.5
LCA-3S	y = 6.52567 x + 0.42518	0.9937	0.2
CDCA-3Gln	y = 0.32583 x - 0.01037	0.9956	0.2
3β-UDCA	y = 0. 12495 x - 0.00731	0.9954	0.5
3-DHCA	y = 0. 16857 x - 0.00152	0.9933	0.2
CDCA-24A-βGlu	y = 0. 18441 x - 0.00921	0.9979	0.5
12-KCDCA	y = 0. 16830 x + 0.00532	0.9960	2.0
7，12-DKLCA	y = 0.02724 x - 0.00288	0.9954	2.0
DHCA	y = 0.00415 x - 0.00182	0.9948	1.0
UCA	y = 0.08117 x - 0.00300	0.9936	0.5
7-DHCA	y = 0.06455 x - 0.00120	0.9935	0.5
isoDCA	y = 0.09412 x + 0.01160	0.9940	0.5
3β-CA	y = 0. 10604 x + 0.03002	0.9948	0.5
T-ω-MCA	y = 0.07842 x - 0.00441	0.9942	0.2
CA-D4	y = 0.20458 x + 0.00549	0.9962	0.5

Supplementary Table S4. SCFAs standard curve

SCFAs name	regression equation	R^2
Acetic acid	y = 0.218900 x + 0.018721	0.999
Propanoic acid	y = 0.303919 x - 0.003980	0.999
Butyric acid	y = 0.969526 x - 0.001560	0.999
Isobutyric acid	y = 0.710925 x - 0.005264	0.999
Valeric acid	y = 1.120411 x - 0.013448	0.999
Isovaleric acid	y = 1.009010 x - 0.017779	0.999
Hexanoic acid	y = 0.932333 x - 0.007077	0.999
Isohexanoic acid	y = 0.481876 x - 0.007273	0.999
